# Comorbidities for Predicting Progression Independent of Relapse Activity in Multiple Sclerosis Treated With B‐Cell Depletion

**DOI:** 10.1111/ene.70656

**Published:** 2026-06-01

**Authors:** Peter Alping, Anton Öberg Sysojev, Fredrik Piehl, Thomas Frisell

**Affiliations:** ^1^ Division of Clinical Epidemiology, Department of Medicine Solna, Karolinska Institutet Stockholm Sweden; ^2^ Department of Clinical Neuroscience Karolinska Institutet Stockholm Sweden

**Keywords:** machine learning, multiple sclerosis, PIRA, prediction, rituximab

## Abstract

**Background:**

Progression independent of relapse activity (PIRA) has been shown to account for a majority of the disability accumulation in relapsing–remitting multiple sclerosis (RRMS) patients treated with disease‐modifying therapies. While comorbidities are common in MS and linked to disability progression, their role in PIRA remains unclear. We investigated whether comorbidities at treatment initiation could predict PIRA in RRMS patients receiving B‐cell depleting therapy.

**Methods:**

This population‐based cohort study included RRMS patients initiating rituximab between August 2010 and April 2019, without relapses during six‐year follow‐up. PIRA was defined as confirmed disability worsening (Expanded Disability Status Scale). We performed hypothesis‐driven analysis of pre‐specified comorbidities and data‐driven analysis of all ICD‐10 codes from secondary care. Comorbidity‐PIRA associations were assessed using adjusted logistic regression. Predictive performance for elastic net, random forest, XGBoost, and neural networks was evaluated as the area under the curve (AUC) and Brier score through nested cross‐validation.

**Results:**

Among 2837 patients, 563 (20%) experienced PIRA within 6 years. The most prevalent comorbidities were depression/anxiety (36%), hypertension (15%), and headache (8%). No pre‐specified comorbidities were statistically significant predictors of PIRA. Among all diagnosis codes, only neuromuscular bladder dysfunction reached significance after multiple comparison correction. Predictive models demonstrated modest performance (AUC 0.563–0.652) and adding comorbidities did not improve prediction.

**Conclusions:**

In this rituximab‐treated RRMS cohort, comorbidities at therapy start were not associated with higher PIRA risk when accounting for MS‐associated disability. The association with bladder dysfunction likely reflects spinal tract engagement rather than an independent comorbidity effect.

## Graphical abstract

In this population‐based cohort study of 2837 rituximab‐treated relapsing–remitting multiple sclerosis (RRMS) patients, 563 (20%) experienced progression independent of relapse activity (PIRA) over 6 years. No pre‐specified comorbidities predicted PIRA and only neuromuscular bladder dysfunction (N31) reached significance among all ICD‐10 codes. Machine learning models showed modest performance (AUC 0.563–0.652), and adding comorbidities did not meaningfully improve prediction.

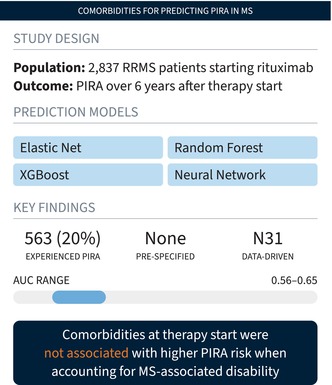

## 1 Introduction

Comorbidities are common in persons with multiple sclerosis (MS) and several, such as inflammatory bowel disease, epilepsy, depression, and anxiety, appear to be more common in persons with MS than in the general population [1]. Some comorbidities have also been linked to general disability progression, with the strongest evidence for depression and epilepsy [2]. However, studies assessing associations between comorbidities and progression independent of relapse activity (PIRA) remain scarce.

Early PIRA, defined as confirmed disability worsening in the absence of clinical relapses, is associated with poor long‐term prognosis [3, 4] and the effectiveness of current disease‐modifying therapies (DMTs) for this aspect of MS remains unclear [5]. The most commonly used DMT for MS in Sweden today, the off‐label B‐cell‐depleting antibody rituximab, is highly effective at reducing clinical relapses and radiological signs of inflammatory activity [6]. However, as persons with relapsing–remitting MS (RRMS) suffer fewer relapses, evidence suggests that a majority of overall disability accumulation occurs through PIRA [3, 7].

The most consistently identified predictor of PIRA is older age, but it has also been associated with T1 and T2 lesion burden, brain atrophy, disease duration, male sex, lower perceived health status, and disability [4, 7, 8]. Given that comorbidities are common in the MS population and have been linked to general disease progression [1, 2], specific comorbidities may also influence the risk of PIRA.

Despite the clinical shift toward PIRA‐dominant progression in patients on highly effective DMTs, no studies have systematically evaluated whether comorbidities predict PIRA in this population. The Swedish setting, with its large cohort of rituximab‐treated MS patients and high‐quality national registers, offers a unique opportunity to assess disease progression in an MS population largely free of relapses and where treatment discontinuation is rare [9].

In this study, we aimed to predict the risk of PIRA, using a standardized definition [10], in RRMS patients receiving rituximab, based on both pre‐specified hypothesis‐driven and data‐driven comorbidity information, to identify risk factors for PIRA, expand the understanding of the impact of comorbidities, and inform personalized treatment strategies in MS. This is the first large‐scale, population‐based investigation of comorbidity‐PIRA associations in a homogeneously treated MS population with minimal confounding from breakthrough disease activity and therapy discontinuation.

## 2 Methods

In this population‐based cohort study, we linked the Swedish MS Register to national health and demographic registers.

### 2.1 Setting and Data Sources

The MS Register is a clinical tool for documenting patient information and contains data on MS characteristics and treatments. While participation in the MS Register is voluntary, all specialist neurology clinics in Sweden are represented, with completeness estimated to 85% [11, 12, 13]. The National Patient Register includes International Classification of Diseases (ICD‐10) codes and dates for all public and private, secondary and inpatient care since 2001, but not primary care [14, 15]. The Prescribed Drug Register includes ATC codes and dates for all dispensations of prescribed drugs since 2005 [16]. The Cancer Register includes all cancer diagnoses in Sweden since 1958 [17, 18]. The Total Population Register includes information on birth, death, migrations, civil status (married/partner), area of residence, and region of origin, for all residents in Sweden [19, 20], while the Income and Taxation Register contains information on annualized earnings from paid work (salaried income) [21] and the Longitudinal Integration Database for Health Insurance and Labor Market Studies (LISA) contains information on highest achieved education level [22, 23].

### 2.2 Study Population

We identified all first‐ever treatments with rituximab in patients with RRMS between 2010‐08‐01 and 2019‐04‐30, which allowed for at least 5 years of look‐back time and 6 years follow‐up from therapy start, given the availability of data. Exclusion criteria were lack of a baseline Expanded Disability Status Scale (EDSS) score (between −1 year and +1 month from therapy start), no EDSS scores during follow‐up (between +1 month and +6 years from therapy start), and relapse, emigration, or death within 6 years of therapy start. Stopping or switching therapy was not considered a censoring event.

### 2.3 Comorbidities

Pre‐specified comorbidities were chosen to include conditions suspected to affect PIRA based on evidence from previous studies and pathomechanistic plausibility (Tables S1 and S2). An alternative, data‐driven approach included a set of binary variables encoding the presence/absence of each unique ICD code truncated to the category level (three characters), as main or contributory diagnosis in inpatient or specialized outpatient care (Table S2). Both pre‐specified and data‐driven comorbidities were identified in the 5 years before therapy start.

### 2.4 Auxiliary Predictors

Demographic variables included age at therapy start, sex, country of birth, annual income (averaged over the last 3 years), highest achieved education (categorized as ≤ 9 years [primary], 10–12 years [secondary], and ≥ 13 years [higher education]), and area of residence in Sweden. MS characteristics included year of therapy start, time since MS onset, number of unique previous DMTs (glatiramer acetate and interferons counted as a single type), number of previous relapses, EDSS, Symbol Digit Modalities Test (SDMT), and Multiple Sclerosis Impact Scale‐29 (MSIS‐29, both the physical and psychological score). Baseline EDSS, SDMT, and MSIS‐29 scores were chosen as the score closest in time to therapy start, within −1 year and +1 month, ignoring any score recorded within 30 days of a relapse.

### 2.5 PIRA

PIRA was defined as proposed by Müller et al. (criteria A3‐B2‐C2‐D4‐E1) [10], with confirmed disability worsening occurring between 1 month and 6 years from therapy start (EDSS scores after 6 years could still be used for confirmation). Disability worsening was defined as a change from baseline EDSS of at least: 1.5 if baseline EDSS was 0, 1.0 if baseline EDSS was 1–5.5, and 0.5 if baseline EDSS was 6–9.5. Re‐baselining occurred after confirmed (≥ 3 months period) EDSS score improvement. A confirmation period of ≥ 12 months was used for EDSS score worsening and all EDSS scores within the confirmation period had to remain at or above the worsening threshold.

### 2.6 Statistics

Associations between individual pre‐specified comorbidities and PIRA were assessed in logistic regression models, both crude (unadjusted) and adjusted for all baseline characteristics including the other pre‐specified comorbidities. For the fully adjusted model, multiple imputation by chained equations was used to impute missing data (20 imputed datasets, 10 burn‐in iterations) and estimates were pooled using Rubin's rules.

Associations between individual ICD categories and PIRA were tested using the Fisher's exact test. ICD categories were excluded from analysis if > 99% of the participants either had or did not have the specific category. The *p* values were visualized in a Manhattan plot on the $-\mathrm{lo}g_{10}$ scale, using a Bonferroni‐corrected significance threshold ($\alpha =\frac{0.05}{\#\text{comparisons}}$).

To assess the predictive power of both the pre‐specified comorbidities and the individual ICD categories, we compared the prognostic capabilities of four different types of predictive models, using four incrementally inclusive sets of predictors. The models were elastic net (regularized logistic regression), random forest, XGBoost (extreme gradient boosting), and a single‐hidden‐layer neural network. The predictor sets were demographic variables, MS characteristics, pre‐specified comorbidities, and individual ICD categories, where each new predictor set included the predictors from previous sets (Table S2). For each model and predictor set, we reported the area under the receiver operating characteristic (AUC, where 0.5 represents chance and 1.0 represents perfect prediction) and the Brier score, as well as plotted the calibration curve. The calibration curves were constructed by binning the predicted probabilities by quantiles of the predicted probabilities among those who had the outcome.

### 2.7 Pre‐Processing

Before fitting each model, but within the resampling (see below) to avoid data leakage, all numeric predictors were normalized, missing data were imputed, and all nominal predictors were dummy encoded. The imputation step included the outcome and all variables in the specific predictor set, except for individual ICD categories. Imputation was performed using a bagged tree model with 25 trees.

### 2.8 Models and Evaluation

Different models had different hyperparameters, some of which were set to static values while the rest were tuned. The elastic net model had two hyperparameters. The random forest model had three hyperparameters, of which the number of trees was set to 1000. The XGBoost model had five hyperparameters, of which the number of trees was set to 1000 and the stopping iterations to 10. The neural network model had three hyperparameters, of which the number of hidden units was set to 50. For all models, hyperparameter values for use in tuning were specified in a regular grid with four levels, where each combination of hyperparameter values was assessed in the tuning. The tuned hyperparameters and their potential values are shown in Table S3. Predictive performance was evaluated through cross‐validation (10 folds, 3 repeats), with hyperparameter tuning in a nested cross‐validation (5 folds), a rigorous validation approach that prevents overfitting. Hyperparameter tuning was optimized for the Brier score.

### 2.9 Sensitivity Analyses

We performed sensitivity analyses for a subpopulation aged 50 years or older at the start of treatment to assess the influence of age on comorbidity patterns and their associations with PIRA. We also visualized change in EDSS (excluding scores recorded within 30 days of a relapse) stratified by whether the person developed PIRA or not during the follow‐up to assess whether PIRA status reflected increasing EDSS as expected, as well as the proportion still on rituximab over time. Finally, we assessed whether the time a person was exposed to a comorbidity affected the results by restricting the pre‐defined comorbidities to those with at least 3 years of exposure time at therapy start.

### 2.10 Software

Data processing and visualization were performed with Python 3.13 and modeling and analysis with R 4.5.0. The prediction modeling pipeline relied heavily on the Tidymodels R packages, using resample for the cross‐validation resamples and recipes for the pre‐processing of data. The models were used through the parsnip integration layer and included glm for elastic net, ranger for random forest, xgboost for XGBoost, and nnet for the neural network. Pre‐processing imputation used step_impute_bag from the recipes R package, while multiple imputation was performed using the mice R package.

## 3 Results

A total of 2837 persons with RRMS starting rituximab treatment fulfilled the inclusion criteria (Table S4), of which 563 (20%) experienced PIRA within 6 years of therapy start.

Mean age at therapy start was 40 years, with 71% of participants being female. Median time since MS onset was 7.0 years, year of therapy start 2016, number of previous DMTs 1, and number of previous relapses 2 (Table 1). Mild disability (EDSS 0–2.5) was most common at 75%, followed by moderate (3–4.5) at 20% and severe (5–9.5) at 5%. The largest differences in baseline characteristics between those who later developed PIRA and those who did not were in age (mean 42 vs. 40), EDSS (more mild vs. more moderate/severe), SDMT (median 52 vs. 54), and MSIS‐29 Physical/Psychological (median 2.0/2.2 vs. 1.4/2.0). Missing data primarily affected baseline SDMT (28%) and MSIS‐29 (28%, Table S5). More than 70% of the study population remained treated with rituximab at the end of the six‐year follow‐up period (Figure S1).

**TABLE 1:** Baseline characteristics for the study population.

Variable	All	No PIRA	PIRA	SMD
*N*	2837	2274	563	—
Age, years	40.4 (10.6)	39.9 (10.6)	42.0 (10.8)	0.138
Sex, female	2025 (71.4)	1621 (71.3)	404 (71.8)	0.007
Born in Sweden	2438 (86.0)	1967 (86.5)	471 (83.7)	−0.057
Married/Partner	1180 (41.7)	947 (41.8)	233 (41.5)	−0.003
Income (thousand SEK)	209.3 (64.0–335.6)	219.6 (69.1–340.1)	172.4 (44.5–318.1)	−0.085
Education, ≤ 9 years	249 (8.8)	197 (8.7)	52 (9.2)	0.014
Education, 10–12 years	1272 (44.8)	1014 (44.6)	258 (45.8)	0.018
Education, ≥ 13 years	1279 (45.1)	1034 (45.5)	245 (43.5)	−0.028
Education, missing	37 (1.3)	29 (1.3)	8 (1.4)	0.009
Years since onset	7.0 (2.6–13.4)	6.9 (2.5–13.0)	7.3 (2.8–13.7)	0.05
Year of therapy start	2016 (2015–2017)	2016 (2015–2018)	2016 (2014–2017)	−0.163
Number of previous DMTs	1.0 (1.0–2.0)	1.0 (1.0–2.0)	1.0 (1.0–2.0)	0.022
Number of previous relapses	2.0 (1.0–3.0)	1.0 (1.0–3.0)	2.0 (1.0–3.0)	0.016
EDSS, mild	2124 (74.9)	1758 (77.3)	366 (65.0)	−0.194
EDSS, moderate	579 (20.4)	430 (18.9)	149 (26.5)	0.128
EDSS, severe	134 (4.7)	86 (3.8)	48 (8.5)	0.14
SDMT	54.0 (46.0–62.0)	54.0 (47.0–63.0)	52.0 (44.0–59.0)	−0.193
MSIS‐29 Physical	1.5 (1.1–2.2)	1.4 (1.1–2.1)	2.0 (1.4–2.8)	0.377
MSIS‐29 Psychological	2.0 (1.4–2.9)	2.0 (1.4–2.8)	2.2 (1.7–3.1)	0.212
Area, Stockholm	968 (34.1)	737 (32.4)	231 (41.0)	0.127
Area, East Middle Sweden	439 (15.5)	357 (15.7)	82 (14.6)	−0.022
Area, South Sweden	219 (7.7)	176 (7.7)	43 (7.6)	−0.003
Area, North Middle Sweden	272 (9.6)	231 (10.2)	41 (7.3)	−0.072
Area, Middle Norrland	156 (5.5)	131 (5.8)	25 (4.4)	−0.042
Area, Upper Norrland	265 (9.3)	218 (9.6)	47 (8.3)	−0.031
Area, Småland and the islands	115 (4.1)	95 (4.2)	20 (3.6)	−0.023
Area, West Sweden	395 (13.9)	323 (14.2)	72 (12.8)	−0.029
Area, missing	8 (0.3)	6 (0.3)	2 (0.4)	0.012

*Note:* Number (proportion in percent), mean (standard deviation), or median (interquartile range). EDSS categorized as mild (0–2.5), moderate (3–4.5), or severe (5–9.5).

Abbreviations: DMT, disease modifying therapy; EDSS, Expanded disability status scale; MSIS‐29, multiple sclerosis impact scale; SDMT, symbol digit modalities test; SMD, standardized mean difference.

**FIGURE 1:**
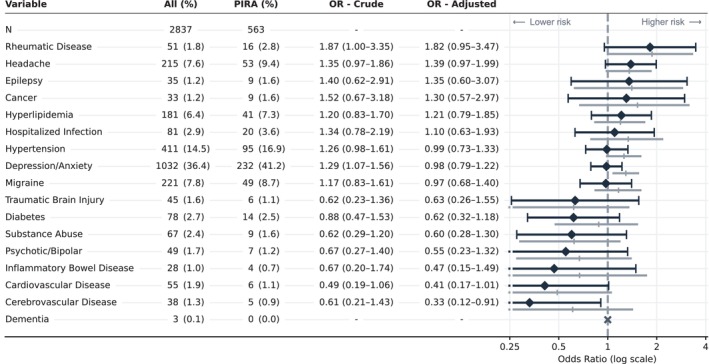
Pre‐specified comorbidities and their association to progression independent of relapse activity (PIRA). Number (proportion in percent) of patients with the pre‐specified comorbidity in the 5 years before therapy start (not including the index date), for the entire study population (All) and those who experienced PIRA. Odds ratios (OR), with 95% confidence intervals, for the associations between the different pre‐specified comorbidities and PIRA, in a model with only the specific comorbidity (Crude) and in a model with all baseline characteristics, including the comorbidities (Adjusted). The forest plot depicts the OR and 95% confidence intervals for each comorbidity from both the crude model (gray, shifted down) and the fully adjusted model (black).

The most common pre‐specified comorbidities in the five‐year period before therapy start were depression/anxiety (36%), hypertension (15%), headache (8%), migraine (8%), and hyperlipidemia (6%, Figure 1). The comorbidities with the strongest evidence for an association with PIRA, after fully adjusting for baseline characteristics, were headache (odds ratio [OR] 1.39, 95% confidence interval [CI] 0.97–1.99) and rheumatic disease (1.82, 0.95–3.47). Cardiovascular and cerebrovascular disease were both associated with a lower risk of PIRA (0.41, 95% CI 0.17–1.01 and 0.33, 0.12–1.91, respectively), but with large uncertainty due to few events.

**FIGURE 2:**
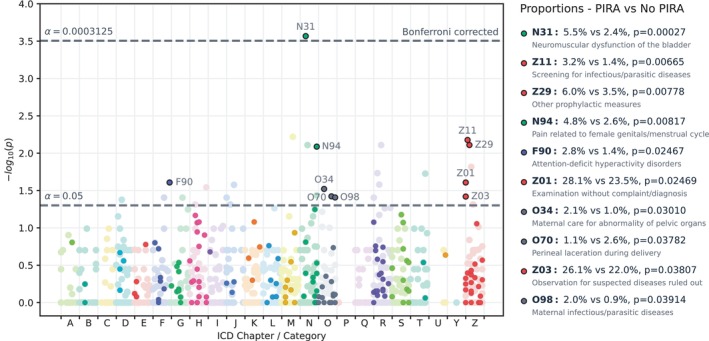
Manhattan plot of the individual International Classification of Disease (ICD) categories. Each colored dot represents the *p* value from a Fisher's exact test, on the $-\mathrm{lo}g_{10}$ scale, that the underlying distributions are the same between those who would develop progression independent of relapse activity (PIRA) and those who would not, for that particular ICD category. ICD categories were excluded if > 99% of the observations either had or did not have the specific category (shown in lighter color). The lower dashed line shows the standard significance threshold of α=0.05, while the upper dashed line shows the significance threshold after Bonferroni correction. ICD categories with a *p* value below the standard significance threshold are indicated with an outline and a label, with proportions and *p* values shown to the right of the plot.

Among all ICD categories in the data‐driven approach, only N31 (neuromuscular dysfunction of the bladder, present in 5.5% vs. 2.4% of the PIRA and non‐PIRA groups, respectively) reached statistical significance after Bonferroni correction (Figure 2). ICD categories with *p* values below the standard significance level (α=0.05) were N94 (4.8% vs. 2.6%, pain related to female genitals/menstrual cycle), F90 (2.8% vs. 1.4%, attention‐deficit hyperactivity disorders), and different O (pregnancy, childbirth, and the puerperium) and Z (factors influencing health status and contact with health services) categories.

**FIGURE 3:**
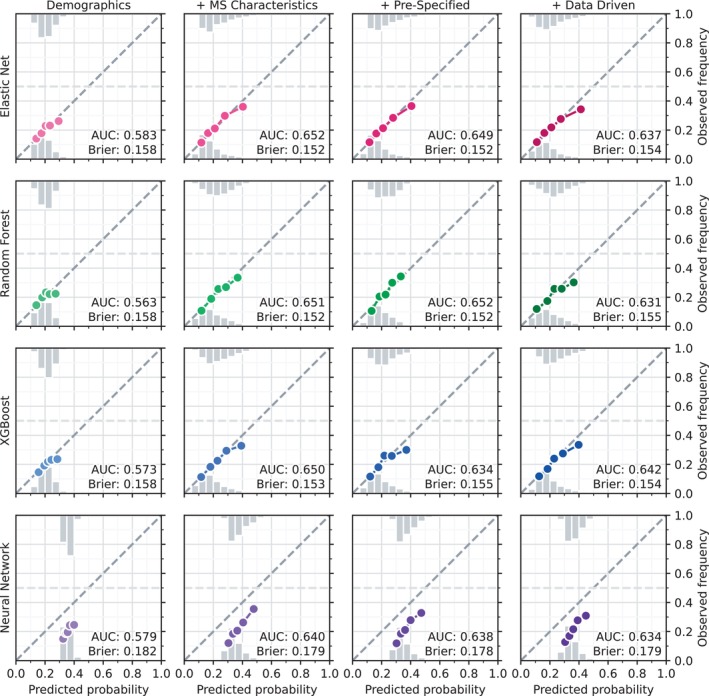
Calibration curves. Calibration of the predicted probabilities, relative to the observed proportions, of progression independent of relapse activity (PIRA) for the different models and predictor sets. Each incremental predictor set included all the variables from the previous sets. Colored dots show the proportions with PIRA by predicted probability binned in quantiles of the predicted probabilities among those with PIRA. The dashed line represents optimal calibration. Histograms show distributions of predicted probabilities for those with PIRA (top) and those without PIRA (bottom). Demographics: Age at therapy start, sex, if the person was born in Sweden, annual income, highest achieved education, and region in Sweden. MS Characteristics: Year of therapy start, time since MS onset, number of unique previous DMTs, number of previous relapses, EDSS, SDMT, and MSIS‐29. Pre‐specified: Cancer, cardiovascular disease, cerebrovascular disease, dementia, depression/anxiety, diabetes, epilepsy, headache, hospitalized infection, hyperlipidemia, hypertension, inflammatory bowel disease, migraine, psychotic/bipolar, rheumatic disease, substance abuse, and traumatic brain injury. Data driven: Individual ICD codes truncated to the category level. AUC, Area Under the Curve for the receiver operating characteristic, equivalent to the c‐index for a binary outcome; Brier, Brier Score.

When predicting PIRA status, all predictor sets (Table S2) and models (Table S3), except the neural network, were well calibrated (predicted probabilities close to the observed frequencies of the outcome), but with the majority of the predicted probabilities below 0.5 (indicative of no strongly predictive features, Figure 3). The AUC, measuring discriminative performance, ranged from 0.563 (worst; Random Forest with only Demographics) to 0.652 (best; both Elastic Net with MS Characteristics and Random Forest with Pre‐Specified Comorbidities). For most models, the discriminative performance increased when adding MS characteristics to the demographic features, remained steady or decreased slightly when adding the pre‐specified comorbidities, and decreased when adding individual ICD categories, except for XGBoost, which performed the worst with the Pre‐Specified Comorbidities predictor set. The Brier score, of which one component measures calibration, followed the same pattern as the AUC and ranged from 0.182 (worst; Neural Network with only Demographics) to 0.152 (best; both Elastic Net and Random Forest with MS Characteristics/Pre‐Specified Comorbidities).

In the sensitivity analysis of the subpopulation aged 50 years or older at the start of treatment, a total of 687 persons were included, of whom 131 (24%) experienced PIRA within the follow up. As expected, most comorbidities were more common in this subpopulation, with hypertension and hyperlipidemia increasing the most (Figure S2). The smaller cohort size resulted in larger confidence intervals, making the odds ratios difficult to interpret. Among the ICD categories, none reached statistical significance after Bonferroni correction, but N31 was still the category with the most evidence of an association (Figure S3). The subgroup was too small for meaningful prediction modeling. Finally, the sensitivity analysis requiring at least 3 years of comorbidity exposure at the time of therapy start reduced the number of persons with each comorbidity, introducing more uncertainty in the estimates, but was otherwise consistent with the main results, except for rheumatic disease, which went from a borderline positive association to no apparent association (Figure S4).

Individuals who developed PIRA during follow‐up increased more in EDSS over time, compared with those who did not develop PIRA, indicating that the PIRA definition indeed reflects progression as measured by EDSS (Figure S1).

## 4 Discussion

We leveraged a large nationwide cohort of rituximab‐treated RRMS patients to explore if comorbidities present at treatment start predicted future PIRA, but found only limited added value on top of demographics and MS characteristics. Inclusion of the full range of individual ICD categories, in a data‐driven approach, generally made predictions worse than with a pre‐specified selection of comorbidities.

Although previous studies of associations between comorbidities and PIRA are scarce, studies of general disease progression in MS have reported links to depression and epilepsy [1, 2]. Several cohort studies found that depression or early depressive symptoms were associated with reaching higher EDSS scores (usually EDSS ≥ 6) [24, 25, 26, 27]. However, in one case, the association diminished when adjusting for baseline EDSS [26], while another study found no association with disease progression at all [28]. Epilepsy has similarly been associated with reaching higher EDSS scores in large cohort studies [29, 30], while epileptic seizures as a first symptom of MS did not seem to affect the disease course [30]. Epilepsy has also been associated with an increased Multiple Sclerosis Severity Score (MSSS) [31].

We did not find any evidence that these comorbidities were predictors of PIRA in our cohort. This is not unexpected given the conflicting evidence for general disease progression, but may also reflect the assumed difference in pathomechanisms for general disease progression, which includes relapse‐associated worsening, compared with PIRA measured in a relapse‐free MS population. Further, the association between depression and PIRA was attenuated when we adjusted for demographics and MS characteristics, similar to the study by Jacobs et al. [26], indicating that depression might be a result of disability rather than a risk factor for it.

We found borderline significant signals of higher odds of PIRA for rheumatic disease and headache, and lower odds for cardiovascular and cerebrovascular disease. These may reflect chance findings, but speculatively, the higher odds for rheumatic disease could be an indicator of a general inflammatory tendency, which in turn could be a risk factor for PIRA, while the higher odds of PIRA among those diagnosed with headache may be a result of headache being a symptom of the underlying process that also causes PIRA, sometimes called “smoldering” MS [32]. Cardiovascular and cerebrovascular disease both had few observed events, resulting in wide confidence intervals, and it is unlikely that these conditions would be protective in a causal sense even if a true association was observed. It is also likely that symptoms believed to be caused by a previous cerebrovascular event are excluded from the EDSS scoring, thereby raising the bar for PIRA.

Among all ICD categories, only N31 (neuromuscular dysfunction of the bladder) stood out as significantly differently distributed between those who later developed PIRA and those who did not, after Bonferroni correction of the significance threshold. This diagnosis could indicate greater spinal involvement related to PIRA, and is likely directly linked to MS disability and the baseline EDSS score (which is a predictor of PIRA). Bladder dysfunction has previously been associated with a higher risk of incomplete recovery after relapses [33], which, together with our findings, suggests that bladder dysfunction is a marker of diminished reserves in the long tracts of the spinal cord.

Sensitivity analyses in an older subpopulation showed overall higher levels of comorbidity, but the smaller cohort size made interpretations of the analyses difficult. However, the pattern of comorbidities was similar, and N31 was again identified as the ICD category with the most evidence for an association. The analysis restricting comorbidities to at least 3 years of exposure at therapy start was mostly consistent with the main analysis, with the exception of an attenuated association between PIRA and rheumatic disease. Notably, a similar attenuation did not affect the association with headache, the only other borderline significant positive association.

Our results indicate that most of the predictive power could be found within the MS characteristics, possibly because these are better than comorbidities at capturing the disease‐specific processes that underlie PIRA or are part of the definition of PIRA, as is the case for EDSS.

### 4.1 Strengths and Limitations

The main strength of our study is that we were able to leverage a large‐scale MS population with active disease at treatment initiation, but with few or no new relapses during follow‐up, which should minimize the risk of PIRA misclassification due to relapse‐associated worsening, especially considering the lack of rebound disease activity when extending rituximab infusion intervals [34]. Sweden is unique in that rituximab has been the most frequently used MS therapy for a decade, making the current study population representative of the larger MS population, with the benefit of lower drug switching and relapse rates compared with other drug classes [35, 36]. This allowed us to complement the pre‐specified comorbidity definitions with a data‐driven approach to identify comorbidity patterns associated with the risk of PIRA through machine learning models, with the capacity to discover non‐linear associations and interactions among the predictors in a researcher‐agnostic way. The use of a standardized definition of PIRA improved the robustness of our outcome and allowed for comparability with future studies.

The main limitation of our study concerns the modeling of PIRA. PIRA is often defined as a change in EDSS score from a baseline, where scores close to relapses have been excluded [10, 37]. Despite its widespread use, EDSS is known to be a crude measure of MS disability [38, 39], which is likely made worse by the dichotomization of EDSS‐change into disability worsening. The clinical relevance of PIRA and the lack of alternative definitions justify this modeling choice, and the use of a standardized PIRA definition improves comparability. Further, coding comorbidities with a set of binary predictors generated from ICD categories is a simplification of a person's medical history and we had no information from primary care. For conditions where most patients would be followed in primary care (such as depression), we lessen the impact of this by using prescribed drugs as an indicator of disease for the pre‐specified comorbidities. The worse performance of the neural network, compared with the other models, is likely in part due to its simple implementation as a feed‐forward single‐hidden‐layer neural network with only 50 nodes in the hidden layer. Developing a more advanced neural network model was beyond the scope of this study and a potential next step for further exploring covariate associations with PIRA. However, this would likely require a much larger study population. We also do not yet know enough about modern DMTs and PIRA to be able to say with certainty that any factors associated with PIRA in persons treated with B‐cell‐depleting therapy are the same as for those treated with other types of DMTs. Despite the significant strengths of the Swedish setting, with its large population of anti‐CD20‐treated MS patients and rich data sources, it could limit generalizability to other contexts. However, the most common comorbidities in our study population closely mirror what has previously been found in studies of other MS populations [1], and reflect the ailments that are endemic to Western society, indicating reasonable generalizability. Finally, although our population represents one of the largest MS cohorts studied to date on a treatment effective enough to suppress relapse‐associated worsening and permit uninterrupted follow‐up, the number of events was too low for meaningful inference for some of the pre‐specified comorbidities. Most notably for dementia, where we found no events, but also for cerebrovascular disease, cardiovascular disease, inflammatory bowel disease, and cancer, largely reflecting the younger age of an actively treated MS population.

## 5 Conclusions

In this large nationwide cohort study of rituximab‐treated RRMS patients, we found no evidence that specific comorbidities predict PIRA, beyond associations through MS‐related disability. Further inclusion of the full range of individual ICD categories in a data‐driven, machine learning approach generally made the predictions of PIRA worse than when restricting to a pre‐specified selection of comorbidities based on prior clinical considerations. Collectively, this indicates that broad comorbidity data available at treatment initiation do not provide strong predictive information of later PIRA, with the possible exception of rheumatic disease. Future work on predictors of PIRA may be more successful if focused on MS‐specific factors rather than co‐occurring medical conditions. Finally, our results suggest that proper management of comorbidities in MS, although likely important in its own right, might not lead to reduced PIRA. However, more research on this topic is needed.

## Author Contributions

All authors took part in the conception and design of the study and in drafting the manuscript. Peter Alping and Thomas Frisell acquired and analyzed the data.

## Funding

This work was supported by a US Department of Defense Early Investigator Research Award (HT9425‐25‐1‐0689, MS240289) and grants from Märta Lundqvists Stiftelse, Petrus och Augusta Hedlunds Stiftelse (M‐2025‐2658), MS Forskningsfonden, Karolinska Institutet, the Swedish Research Council (2023‐02639, 2021‐01418 and 2024‐02744), Region Stockholm (FoUI‐987565), Hjärnfonden (FO2023‐0336), and the Erling‐Persson Foundation. The funders of the study had no role in study design, data collection, data analysis, interpretation of the results, or writing of the report.

## Ethics Statement

This study was approved by the Swedish Ethical Review Authority (reference: 2021‐02384 and 2023‐07906‐02). As this is an observational study using already collected data, the ethics board determined that participant consent was not required.

## Conflicts of Interest

Peter Alping and Anton Öberg Sysojev have nothing to report. Thomas Frisell is partly supported by the Anti‐Rheumatic Therapies in Sweden (ARTIS) project, which in turn has been funded by agreements with Abbvie, BMS, Eli Lilly, Pfizer, Roche, Samsung Bioepis, and Sanofi. Fredrik Piehl has received research grants from Denka, Janssen, Merck KGaA, Novartis, Pfizer, and UCB, and fees for serving on DMC in clinical trials with Lundbeck and Roche, and preparation of expert witness statement for Novartis.

## Supporting information


**Table S1:** Comorbidity criteria.
**Table S2:** Variables included in the different predictor sets.
**Table S3:** Hyperparameter values used in tuning.
**Table S4:** Inclusion and exclusion criteria.
**Table S5:** Missing data.
**Figure S1:** Mean EDSS and proportion still on rituximab during follow‐up.
**Figure S2:** Sensitivity Analysis—Forest plot for those aged ≥ 50 years.
**Figure S3:** Sensitivity Analysis—Manhattan plot for those aged ≥ 50 years.
**Figure S4:** Sensitivity Analysis—Forest plot for comorbidities with at least.

## Data Availability

The data are available from the respective register holders. Data will be shared upon reasonable request and within the limits of applicable legislation.
