# Supplementary material for: Comorbidities for Predicting Progression Independent of Relapse Activity in Multiple Sclerosis Treated With B‐Cell Depletion
Source: Eur J Neurol. 2026 Jun 1;33(6):e70656. doi: 10.1111/ene.70656 (PMC13239264; doi:10.1111/ene.70656)
Supplement: Supplementary file 1 — Table S1: Comorbidity criteria. Table S2: Variables included in the different predictor sets. Table S3: Hyperparameter values used in tuning. Table S4: Inclusion and exclusion criteria. Table S5: Missing data. Figure S1: Mean EDSS and proportion still on rituximab during follow‐up. Figure S2: Sensitivity Analysis—Forest plot for those aged ≥ 50 years. Figure S3: Sensitivity Analysis—Manhattan plot for those aged ≥ 50 years. Figure S4: Sensitivity Analysis—Forest plot for comorbidities with at least. [file ENE-33-e70656-s001.docx]

# Supplemental Material

## Comorbidities for Predicting Progression Independent of Relapse Activity in Multiple Sclerosis treated with B-cell Depletion

Peter Alping, MD, PhD^1^, Anton Öberg Sysojev, PhD^1^, Fredrik Piehl, MD, PhD^2^, and Thomas Frisell, PhD^1^

^1^Division of Clinical Epidemiology, Department of Medicine, Solna, Karolinska Institutet, Stockholm, Sweden
^2^Department of Clinical Neuroscience, Karolinska Institutet, Stockholm, Sweden

Peter Alping - 0000-0002-4710-6326 - peter.alping@ki.se
Anton Öberg Sysojev - 0000-0003-3221-7979 - anton.oberg.sysojev@ki.se
Fredrik Piehl - 0000-0001-8329-5219 - fredrik.piehl@ki.se
Thomas Frisell - 0000-0002-5735-9626 - thomas.frisell@ki.se

**Corresponding author:** Peter Alping (peter.alping@ki.se) - Maria Aspmans gata 30A, 171 64, Solna, SWEDEN

### Table of Contents

- [**Supplemental Table 1**](#stbl-comorbidity)**:** Comorbidity criteria
- [**Supplemental Table 2**](#stbl-predictor-sets)**:** Variables included in the different predictor sets
- [**Supplemental Table 3**](#stbl-hyperparameters)**:** Hyperparameter values used in tuning
- [**Supplemental Table 4**](#stbl-inclusion)**:** Inclusion and exclusion criteria
- [**Supplemental Table 5**](#stbl-missing)**:** Missing data
- [**Supplemental Figure 1**](#sfig-follow-up)**:** Mean EDSS and proportion still on rituximab during follow-up
- [**Supplemental Figure 2**](#sfig-forest-above-50)**:** Sensitivity Analysis - Forest plot for those aged ≥50 years
- [**Supplemental Figure 3**](#sfig-manhattan-above-50)**:** Sensitivity Analysis - Manhattan plot for those aged ≥50 years
- [**Supplemental Figure 4**](#sfig-forest-comorb-3y)**:** Sensitivity Analysis - Forest plot for comorbidities with at least three years of exposure time

| **Supplemental Table 1: Comorbidity criteria**   \| **Comorbidity** \| **ICD** \| **ATC** \| **eCode** \| **Note** \| \| --- \| --- \| --- \| --- \| --- \| \| Cancer \| - \| - \| - \| The cancer register (ben ≠ 3) \| \| Cardiovascular Disease \| I (20-25) \| - \| - \| - \| \|  \| I (44\|45\|47-49) \|  \|  \|  \| \|  \| I (110\|130\|132\|255\|420\|426-429\|43\|50) \|  \|  \|  \| \| Cerebrovascular Disease \| G45 \| - \| - \| - \| \|  \| I (60-64\|67\|69) \|  \|  \|  \| \| Dementia \| F (00-03\|051) \| - \| - \| - \| \|  \| G (30\|311\|319) \|  \|  \|  \| \| Depression/Anxiety \| F (32-34\|38\|39\|40\|41) \| N05B \| - \| - \| \|  \|  \| N06A (B\|F\|G) \|  \|  \| \|  \|  \| N06AX (01-19\|22-62) \|  \|  \| \| Diabetes \| E (10-14) \| A10 \| - \| - \| \| Epilepsy \| G (40\|41) \| - \| - \| - \| \| Headache \| G44 \| - \| - \| - \| \|  \| R51 \|  \|  \|  \| \| Hospitalized infection \| A (00-32\|34-49\|51-99) \| - \| - \| Only main diagnosis in inpatient care \| \|  \| B (00-99) \|  \|  \|  \| \|  \| D (733) \|  \|  \|  \| \|  \| E (321) \|  \|  \|  \| \|  \| G (00-02\|042\|05-07) \|  \|  \|  \| \|  \| H (000\|440\|600-603\|66\|67\|70) \|  \|  \|  \| \|  \| I (301\|400) \|  \|  \|  \| \|  \| J (00-22\|32\|340\|36\|390\|391\|440\|85\|86) \|  \|  \|  \| \|  \| K (044\|046\|047\|102\|113\|122\|140\|570\|572\|574\|578\|61\|630\|650-652\|659) \|  \|  \|  \| \|  \| L (00-08\|303) \|  \|  \|  \| \|  \| M (00\|01\|462-465\|600\|650\|710\|711\|726\|86) \|  \|  \|  \| \|  \| N (136\|151\|159\|300\|308\|340\|412\|431\|452-454\|482\|61\|70\|73\|751) \|  \|  \|  \| \| Hyperlipidemia \| E (780\|782\|784\|785) \| C10 \| - \| - \| \| Hypertension \| I (10-13\|15) \| C (02\|03\|07-09) \| - \| - \| \| Inflammatory Bowel Disease \| K (50\|51) \| - \| - \| - \| \| Migraine \| G43 \| N02C \| - \| - \| \| Psychotic/Bipolar \| F (20-29) \| - \| - \| - \| \|  \| F (30-31) \|  \|  \|  \| \| Rheumatic Disease \| M (05\|06\|070-073\|08\|123\|13\|30\|313-316\|32-34\|350\|351\|353\|45\|46) \| - \| - \| - \| \| Substance Abuse \| F (10-16\|18\|19) \| - \| X (41\|42\|45\|61\|62\|65) \| - \| \|  \|  \|  \| Y (11\|12\|15) \|  \| \|  \|  \|  \| T (40\|51) \|  \| \| Traumatic Brain Injury \| S06 \| - \| - \| - \|   Based on International Classification of Diseases (ICD)-10 codes from all inpatient and outpatient care registered in the National Patient Register as a main or auxiliary diagnosis (unless otherwise stated), including codes for external causes of morbidity and mortality (eCode); Anatomical Therapeutic Chemical (ATC) codes from all dispensed prescription drugs in the Prescribed Drug Register; and/or observations in the Cancer Register. |
| --- | --- | --- | --- | --- | --- | --- | --- | --- | --- | --- | --- | --- | --- | --- | --- | --- | --- | --- | --- | --- | --- | --- | --- | --- | --- | --- | --- | --- | --- | --- | --- | --- | --- | --- | --- | --- | --- | --- | --- | --- | --- | --- | --- | --- | --- | --- | --- | --- | --- | --- | --- | --- | --- | --- | --- | --- | --- | --- | --- | --- | --- | --- | --- | --- | --- | --- | --- | --- | --- | --- | --- | --- | --- | --- | --- | --- | --- | --- | --- | --- | --- | --- | --- | --- | --- | --- | --- | --- | --- | --- | --- | --- | --- | --- | --- | --- | --- | --- | --- | --- | --- | --- | --- | --- | --- | --- | --- | --- | --- | --- | --- | --- | --- | --- | --- | --- | --- | --- | --- | --- | --- | --- | --- | --- | --- | --- | --- | --- | --- | --- | --- | --- | --- | --- | --- | --- | --- | --- | --- | --- | --- | --- | --- | --- | --- | --- | --- | --- | --- | --- | --- | --- | --- | --- | --- | --- | --- | --- | --- | --- | --- | --- | --- | --- | --- | --- | --- | --- | --- | --- | --- | --- | --- | --- | --- | --- | --- | --- | --- | --- | --- | --- | --- | --- | --- | --- | --- | --- | --- | --- | --- | --- | --- | --- | --- |

| **Supplemental Table 2: Variables included in the different predictor sets**   \| **Predictor set** \| **Count** \| **Cumulative count** \| **Variables** \| \| --- \| --- \| --- \| --- \| \| Demographic \| 7 \| 7 \| age_y, sex_female, born_in_sweden, partner, area, income_tsek, education \| \| Multiple Sclerosis \| 8 \| 15 \| years_since_onset, index_year, prev_dmts, prev_relapses, baseline_edss, baseline_sdmt, baseline_msis_physical, baseline_msis_psychological \| \| Pre-Specified \| 17 \| 32 \| depression_anxiety, psychotic_bipolar, substance_abuse, cardiovascular_disease, hypertension, diabetes, hyperlipidemia, cerebrovascular_disease, headache, migraine, epilepsy, dementia, traumatic_brain_injury, rheumatic_disease, inflammatory_bowel_disease, hospitalized_infection, cancer \| \| Data Driven \| 160 \| 192 \| G35, F32, Z29, C50, R42, G37, R11, I10, R55, H81, I25, R52, R13, N92, M51, F43, E03, H49, R20, D35, H53, R51, Z03, G04, R33, H46, I63, N20, R10, F10, M35, E04, D25, N83, R07, J06, T88, S61, M79, N39, N84, G43, S52, Z00, R47, F31, S06, N30, Z92, F41, F33, K44, K92, M54, D50, M16, E66, G40, E11, J18, S82, Z30, T81, J45, F60, Z51, Z48, Z09, M70, R69, K59, F17, Z86, Z01, S60, G47, H43, L71, S42, S01, K21, S92, R29, N31, S93, R53, T78, L50, G44, A09, S83, K57, E78, R00, R74, Z85, Z95, Z53, M20, S63, L98, M50, H02, M48, Z98, K50, Z71, N81, J30, M17, Z96, M75, H47, Z04, Z80, L40, R06, H90, H93, Z72, R32, N76, R87, Z76, M77, N87, N94, M23, D48, R19, H40, H35, H20, H04, D23, M25, N93, G56, R39, Z08, N95, D22, L82, C44, L57, H52, S80, H25, Z12, Z13, H57, R30, I83, L30, R22, R23, U99, H61, H10, H01 \|   The name of the predictor set, the number of variables added, the cumulative variable count, and the names of the included variables. |
| --- | --- | --- | --- | --- | --- | --- | --- | --- | --- | --- | --- | --- | --- | --- | --- | --- | --- | --- | --- | --- |

| **Supplemental Table 3: Hyperparameter values used in tuning**   \| **Parameter** \| **Description** \| **Demographic** \| **+ MS Characteristics** \| **+ Pre-Specified** \| **+ Data Driven** \| \| --- \| --- \| --- \| --- \| --- \| --- \| \| **Elastic Net** \| Regularized logistic regression model \| - \| - \| - \| - \| \| penalty \| Regularization strength \| 1e-10, 2.15443469003189e-07, 0.000464158883361278, 1 \| 1e-10, 2.15443469003189e-07, 0.000464158883361278, 1 \| 1e-10, 2.15443469003189e-07, 0.000464158883361278, 1 \| 1e-10, 2.15443469003189e-07, 0.000464158883361278, 1 \| \| mixture \| Balance between L1 (lasso) and L2 (ridge) penalties \| 0, 0.333333333333333, 0.666666666666667, 1 \| 0, 0.333333333333333, 0.666666666666667, 1 \| 0, 0.333333333333333, 0.666666666666667, 1 \| 0, 0.333333333333333, 0.666666666666667, 1 \| \| **Random Forest** \| Decision tree ensemble model \| - \| - \| - \| - \| \| mtry \| Number of predictors randomly sampled at each split \| 2, 3, 5, 7 \| 2, 6, 10, 15 \| 2, 8, 14, 20 \| 2, 11, 20, 30 \| \| min_n \| Minimum number of data points required in a node for further splitting \| 2, 4, 7, 10 \| 2, 4, 7, 10 \| 2, 4, 7, 10 \| 2, 4, 7, 10 \| \| **XGBoost** \| Gradient boosting decision tree model \| - \| - \| - \| - \| \| mtry \| Number of predictors randomly sampled at each split \| 2, 3, 5, 7 \| 2, 6, 10, 15 \| 2, 8, 14, 20 \| 2, 11, 20, 30 \| \| tree_depth \| Maximum depth of the decision trees \| 3, 4, 5, 6 \| 3, 4, 5, 6 \| 7, 8, 9, 10 \| 7, 9, 12, 15 \| \| learn_rate \| Learning rate controlling how quickly the model fits residuals \| 1e-10, 1e-07, 1e-04, 0.1 \| 1e-10, 1e-07, 1e-04, 0.1 \| 1e-10, 1e-07, 1e-04, 0.1 \| 1e-10, 1e-07, 1e-04, 0.1 \| \| **Neural Network** \| Neural network model \| - \| - \| - \| - \| \| penalty \| Weight decay that adds regularization to prevent overfitting \| 1e-10, 2.15443469003189e-07, 0.000464158883361278, 1 \| 1e-10, 2.15443469003189e-07, 0.000464158883361278, 1 \| 1e-10, 2.15443469003189e-07, 0.000464158883361278, 1 \| 1e-10, 2.15443469003189e-07, 0.000464158883361278, 1 \| \| epochs \| Number of complete passes through the training dataset during training \| 10, 340, 670, 1000 \| 10, 340, 670, 1000 \| 10, 340, 670, 1000 \| 10, 340, 670, 1000 \|   All combinations of hyperparameter values were tested in a regular grid for each model. The hyperparameter set that resulted in the lowest average Brier score among the cross-validation folds were used in the model. | | |
| --- | --- | --- | --- | --- | --- | --- | --- | --- | --- | --- | --- | --- | --- | --- | --- | --- | --- | --- | --- | --- | --- | --- | --- | --- | --- | --- | --- | --- | --- | --- | --- | --- | --- | --- | --- | --- | --- | --- | --- | --- | --- | --- | --- | --- | --- | --- | --- | --- | --- | --- | --- | --- | --- | --- | --- | --- | --- | --- | --- | --- | --- | --- | --- | --- | --- | --- | --- | --- | --- | --- | --- | --- | --- | --- | --- | --- | --- | --- | --- | --- | --- | --- | --- | --- | --- | --- |
| **Supplemental Table 4: Inclusion and exclusion criteria**   \| **Criteria** \| **Sample size** \| **Change** \| \| --- \| --- \| --- \| \| All therapies \| 43376 \| 0 \| \| Rituximab \| 10992 \| -32384 \| \| First therapy of type \| 10695 \| -297 \| \| RRMS at therapy start \| 8774 \| -1921 \| \| In study period (2010-08-01:2019-04-30) \| 4808 \| -3966 \| \| In Sweden at index date \| 4792 \| -16 \| \| Enough EDSS (1 baseline, 1 outcome) \| 3149 \| -1643 \| \| Has ≥6y of follow-up \| 2837 \| -312 \| \| No relapse before 6y \| 2837 \| 0 \|   Each row shows the cumulative sample size after the specified and all previous criteria have been applied, together with the change from the previous row and the proportion of the original population in the study cohort. |  |  |

| **Supplemental Table 5: Missing data.**   \| **Variable** \| **All** \| **No PIRA** \| **PIRA** \| **SMD** \| \| --- \| --- \| --- \| --- \| --- \| \| N \| 2837 \| 2274 \| 563 \| - \| \| Age \| 0 (0.0) \| 0 (0.0) \| 0 (0.0) \| - \| \| Sex \| 0 (0.0) \| 0 (0.0) \| 0 (0.0) \| - \| \| Born in Sweden \| 1 (0.0) \| 1 (0.0) \| 0 (0.0) \| -0.021 \| \| Married/Partner \| 8 (0.3) \| 6 (0.3) \| 2 (0.4) \| 0.012 \| \| Income (thousand SEK) \| 17 (0.6) \| 14 (0.6) \| 3 (0.5) \| -0.008 \| \| Education \| 37 (1.3) \| 29 (1.3) \| 8 (1.4) \| 0.009 \| \| Years since onset \| 36 (1.3) \| 33 (1.5) \| 3 (0.5) \| -0.066 \| \| Year of therapy start \| 0 (0.0) \| 0 (0.0) \| 0 (0.0) \| - \| \| Nr. of prev. DMTs \| 0 (0.0) \| 0 (0.0) \| 0 (0.0) \| - \| \| Nr. of prev. relapses \| 0 (0.0) \| 0 (0.0) \| 0 (0.0) \| - \| \| EDSS \| 0 (0.0) \| 0 (0.0) \| 0 (0.0) \| - \| \| SDMT \| 806 (28.4) \| 651 (28.6) \| 155 (27.5) \| -0.017 \| \| MSIS-29 Physical \| 806 (28.4) \| 649 (28.5) \| 157 (27.9) \| -0.01 \| \| MSIS-29 Psychological \| 805 (28.4) \| 648 (28.5) \| 157 (27.9) \| -0.01 \| \| Area in Sweden \| 8 (0.3) \| 6 (0.3) \| 2 (0.4) \| 0.012 \|   Number of observations and proportion in percent with missing data for the baseline variables. The standardized mean difference for the missingness of each variable was calculated between those who would develop PIRA and those who would not. SMD=Standardized Mean Difference. |
| --- | --- | --- | --- | --- | --- | --- | --- | --- | --- | --- | --- | --- | --- | --- | --- | --- | --- | --- | --- | --- | --- | --- | --- | --- | --- | --- | --- | --- | --- | --- | --- | --- | --- | --- | --- | --- | --- | --- | --- | --- | --- | --- | --- | --- | --- | --- | --- | --- | --- | --- | --- | --- | --- | --- | --- | --- | --- | --- | --- | --- | --- | --- | --- | --- | --- | --- | --- | --- | --- | --- | --- | --- | --- | --- | --- | --- | --- | --- | --- | --- | --- | --- | --- | --- | --- |

| 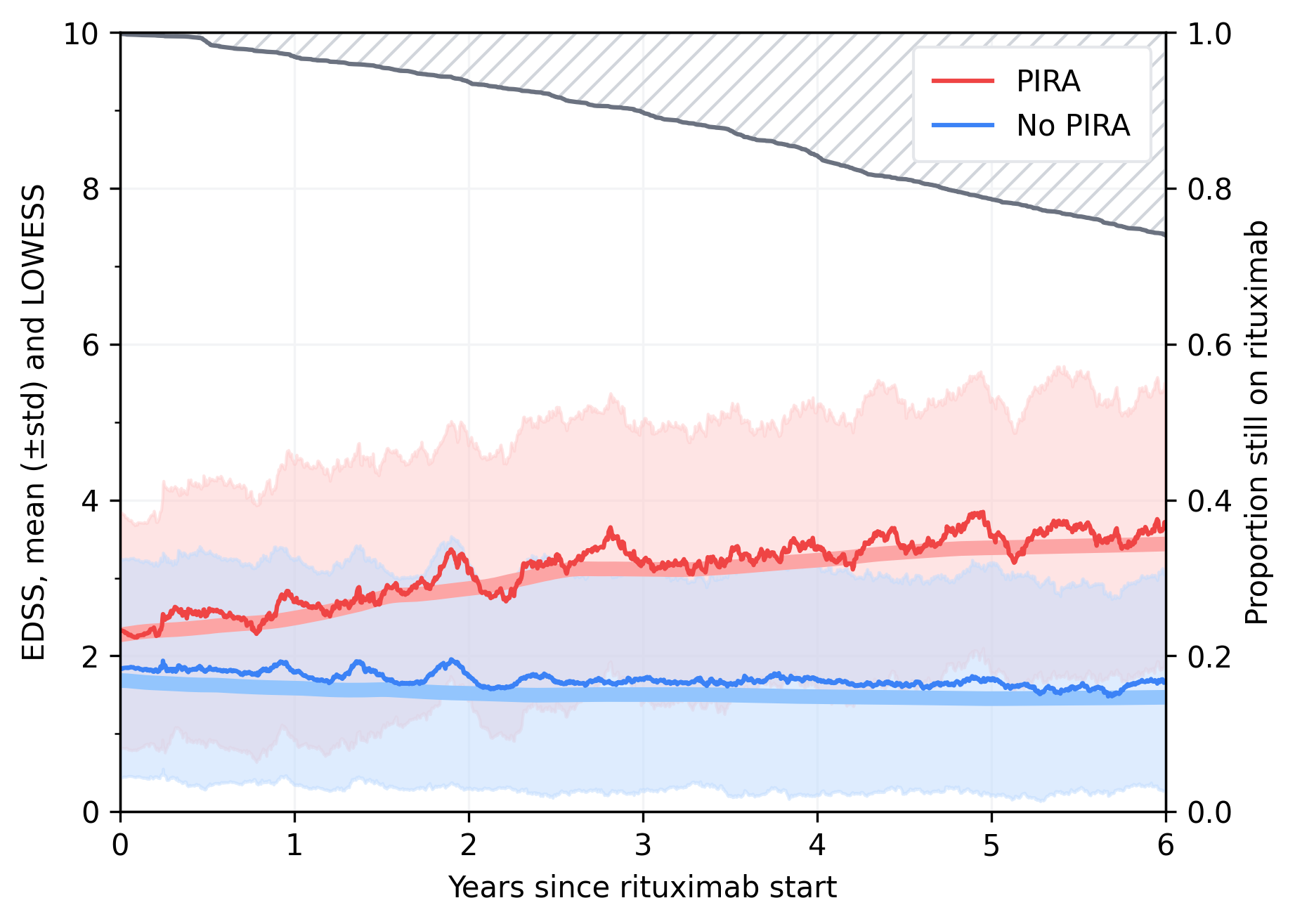  **Supplemental Figure 1: Mean EDSS stratified by PIRA status and proportion still on rituximab during follow-up**  The thinner red (PIRA) and blue (no PIRA) lines show the 90-days cumulative mean EDSS (left vertical axis) over time from rituximab start (horizontal axis), stratified by whether the person developed PIRA during follow-up or not. The shaded colored regions represent plus/minus one standard deviation from the mean and the thicker colored lines are LOWESS estimates of the mean. The gray line shows the proportion of the population still on rituximab (right vertical axis). |
| --- |

| 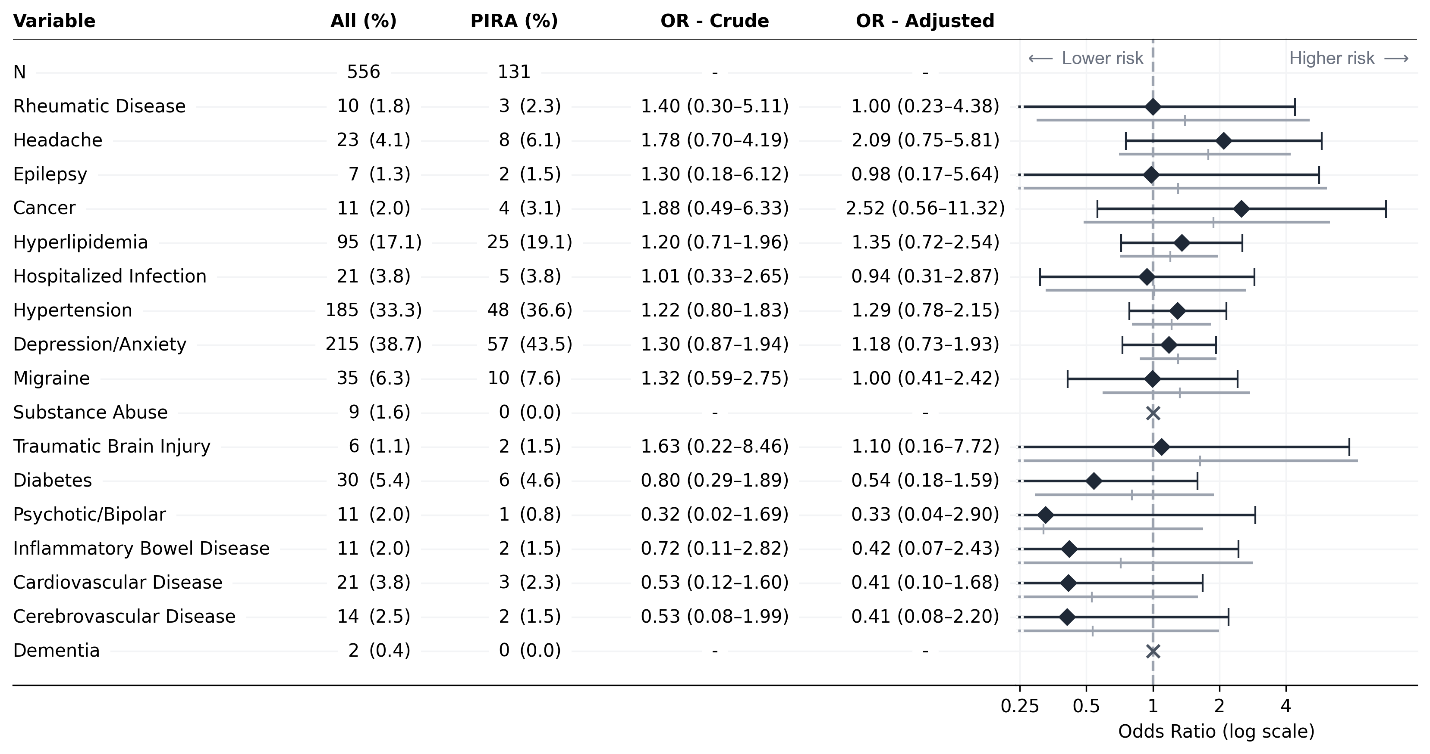  **Supplemental Figure 2: Sensitivity Analysis - Pre-specified comorbidities and their association to progression independent of relapse activity (PIRA) for those aged ≥50 years at treatment start**  Number (proportion in percent) of patients with the pre-specified comorbidity in the five years before therapy start (not including the index date), for the entire study population (All) and those who experienced PIRA. Odds ratios, with 95% confidence intervals, for the associations between the different pre-specified comorbidities and PIRA, in a model with only the specific comorbidity (Crude) and in a model with all baseline characteristics, including the comorbidities (Adjusted). The forest plot depicts the odds ratios (OR) and 95% confidence intervals for each comorbidity from both the crude model (grey, shifted down) and the fully adjusted model (black). |
| --- |

| 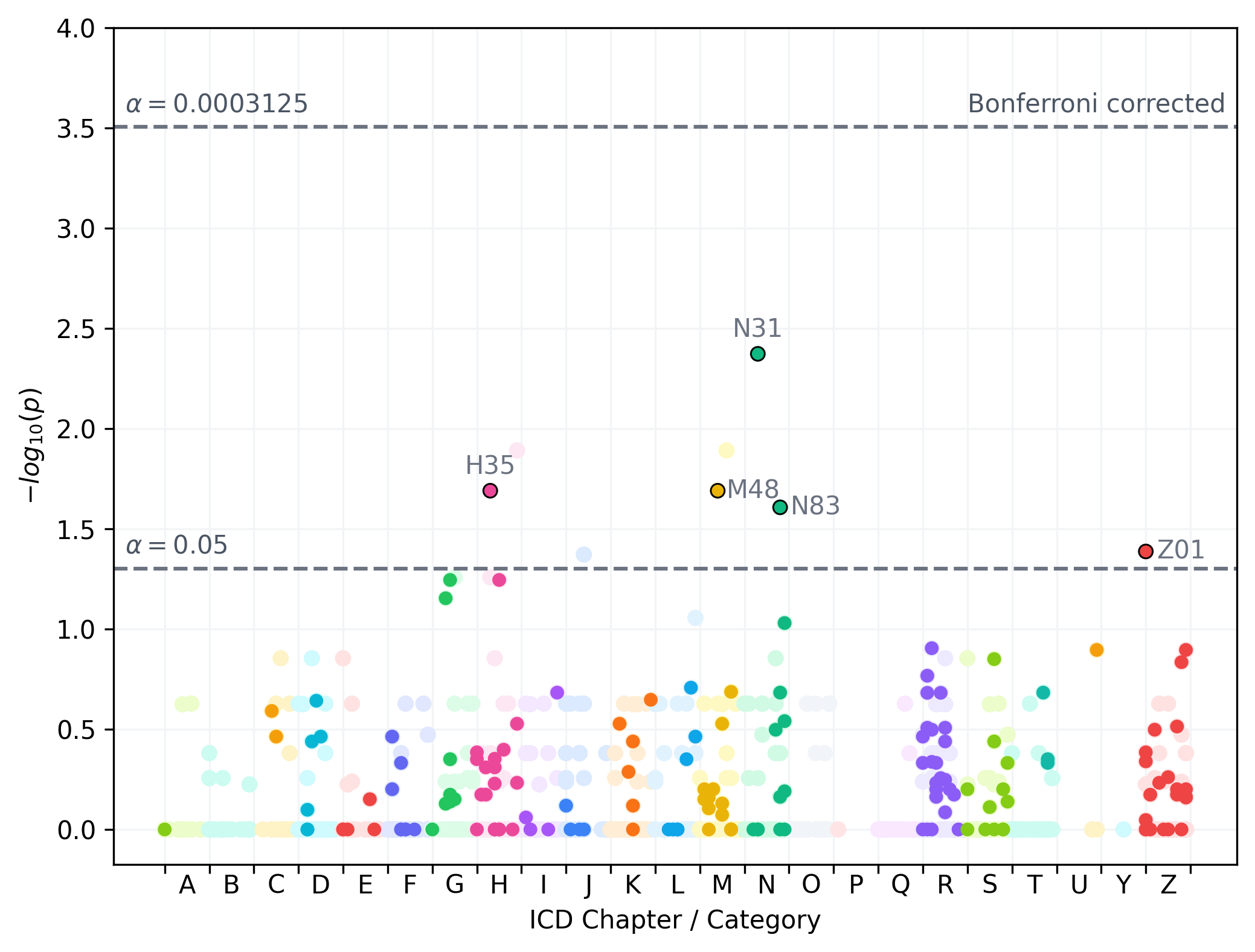  **Supplemental Figure 3: Sensitivity Analysis - Manhattan plot of the individual ICD categories for those aged ≥50 years at treatment start**  Each colored dot represents the p-value from a Fisher’s exact test, on the $-log_{10}$ scale, that the underlying distributions are the same between those who would develop PIRA and those who would not, for that particular ICD category. ICD categories were excluded if >99% of the observations either had or did not have the specific category (shown in lighter color). The lower dashed line shows the standard significance threshold of $\alpha=0.05$, while the upper dashed line shows the significance threshold after Bonferroni correction. ICD categories with a p-value below the standard significance threshold are indicated with an outline and a label, with proportions and p-values shown to the right of the plot. |
| --- |


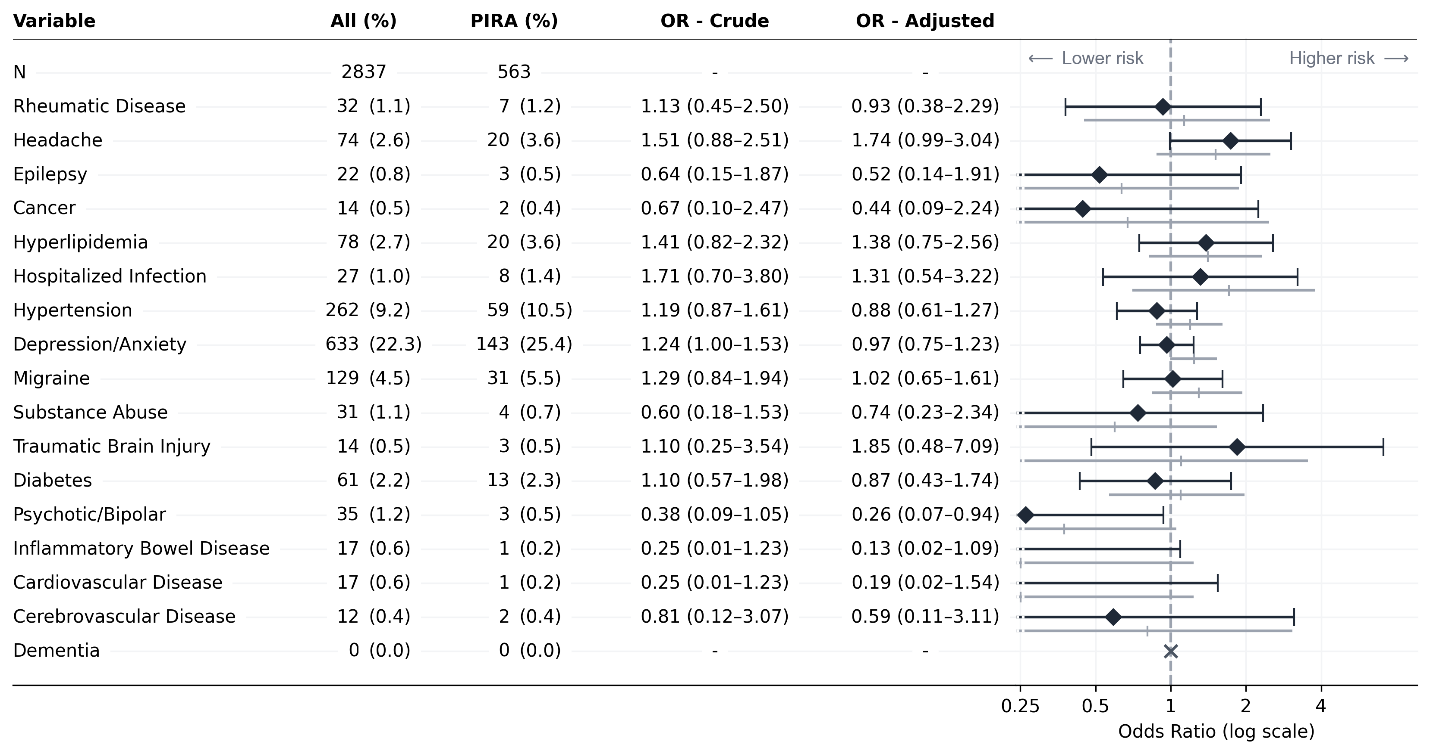


**Supplemental Figure 4: Sensitivity Analysis - Pre-specified comorbidities and their association to progression independent of relapse activity (PIRA) for comorbidities with at least three years of exposure time at treatment start.**

Number (proportion in percent) of patients with the pre-specified comorbidity in the five years before therapy start (not including the index date), for the entire study population (All) and those who experienced PIRA. Odds ratios, with 95% confidence intervals, for the associations between the different pre-specified comorbidities and PIRA, in a model with only the specific comorbidity (Crude) and in a model with all baseline characteristics, including the comorbidities (Adjusted). The forest plot depicts the odds ratios (OR) and 95% confidence intervals for each comorbidity from both the crude model (grey, shifted down) and the fully adjusted model (black).
